# Torque expression in directly printed and thermoformed clear aligners: a controlled in vitro biomechanical comparison of power ridge effects

**DOI:** 10.1186/s40510-026-00647-w

**Published:** 2026-10-07

**Authors:** Aliaa A Khadre, Mohamed Elkorashie, Mostafa Magdy Elzahar, Christoph Bourauel, Ahmed Mahmoud Fouda

**Affiliations:** 1 Department of Orthodontics, Suez Canal University, Ismailia, Egypt; 2 Department of Oral Technology, University Hospital Bonn, Bonn, Germany; 3 Department of Orthodontics, Universitätsmedizin Greifswald, Greifswald, Germany; 4 Department of Fixed Prosthodontics, Suez Canal University, Ismailia, Egypt

**Keywords:** Clear aligners, Direct-printed aligners, Power ridges, Torque control, Orthodontic biomechanics

## Abstract

**Background:**

Torque control of anterior teeth remains a major challenge in clear aligner therapy. Power ridges have been introduced to improve buccolingual root control; however, their biomechanical performance in directly printed aligners remains insufficiently investigated. The aim of this study was to compare the torque efficiency of power ridges in thermoformed and directly printed aligners.

**Methodology:**

A controlled in-vitro study was conducted using a standardised maxillary model requiring incisor torque correction. Four aligner groups were evaluated: Zendura Flex thermoformed aligners (Bay Materials LLC, USA) with (ZF-PR) and without (ZF-C) power ridges, and two directly printed aligners with power ridges fabricated from TA-28 (Graphy Inc., Seoul, South Korea) and DCA (LuxCreo Inc., Bellevue, WA, USA) resin materials. Power ridges were digitally positioned on the labial gingival third of the maxillary central incisors. Biomechanical testing was performed using the Orthodontic Measurement and Simulation System (OMSS) to assess torque expression, root displacement, and force–moment systems under controlled temperatures (25 °C and 37 °C).

**Results:**

Torque expression demonstrated a clear material-dependent pattern. ZF-C, used as the baseline, showed moderate and consistent torque expression (38–39% of the programmed torque) across temperatures, with no significant temperature-dependent differences. DCA exhibited the highest torque expression, reaching 62% at 25 °C and 47% at 37 °C. ZF-PR showed intermediate performance (32–42%) with moderate temperature-related variation. TA-28 demonstrated the lowest torque expression (10–17%) with no significant temperature effect. Secondary outcomes supported these findings, with moment generation and displacement patterns correlating with torque expression, whereas force magnitude did not.

**Conclusions:**

Torque expression in clear aligner systems is primarily governed by material properties and thermomechanical behaviour. Design features alone cannot fully compensate for limitations in material performance. Effective torque delivery depends on the generation and transmission of an appropriate force–moment system rather than on force magnitude alone.

## Introduction and background

Precise torque control of the anterior teeth is a fundamental objective in orthodontics, as it directly affects dental aesthetics, occlusal function, and long-term stability. The buccolingual inclination and anteroposterior position of the maxillary incisors significantly influence smile attractiveness, with mild lingual inclination and slight protrusion generally perceived more favourably than excessive labial inclination or retrusion [1–3]. These findings highlight the critical importance of accurately controlling incisor torque during orthodontic treatment planning and execution [4].

Fixed appliances provide relatively precise torque control. However, achieving comparable root movement with clear aligner therapy remains challenging. Clear aligners depend primarily on material elasticity and aligner–tooth interactions to generate orthodontic forces, often resulting in less predictable buccolingual root movement and reduced torque efficiency compared with fixed appliances [5, 6] leading to incomplete root movement or unintended crown tipping [7].

Clear aligner therapy (CAT) has become an increasingly popular orthodontic treatment modality due to its aesthetic advantages and patient comfort [8]. However, achieving predictable torque expression with clear aligners remains a significant biomechanical challenge [9].

Unlike fixed appliances, which generate torque through a bracket–archwire couple capable of delivering relatively controlled moments, clear aligners produce torque primarily through surface contact between the aligner and tooth, together with deformation of the aligner material. As a result, the generation and maintenance of the moments required for buccolingual root movement are generally less efficient and less predictable than with fixed appliance therapy [9, 10].

In several investigations the predictability of torque expression during CAT has been explored, with particular emphasis on design dependent biomechanical behaviour. In an in vitro analysis, Simon et al. [11] demonstrated that the use of horizontal ellipsoid attachments and power ridges could enhance torque control of anterior teeth, indicating that specific aligner design features may improve torque delivery. Nevertheless, results of clinical studies showed variable outcomes. Gaddam et al. [12] evaluated the effectiveness of Invisalign treatment and observed reduced efficacy in buccal crown torque movement, particularly in maxillary lateral incisors. Similarly, Jiang et al. [13] reported an average torque expression efficiency of approximately 35% during aligner therapy, highlighting the limitations of current aligner systems in achieving accurate root movement [14].

Sandhya et al. [15] employed finite element analysis to evaluate torque movement in maxillary central incisors and found that horizontal ellipsoid attachments produced more effective torque than several alternative attachment designs. Assessing torque effects in vivo is difficult due to the influence of intra- and inter-arch relationships, occlusal contacts, and patient related factors.

To enhance torque delivery in aligner therapy, auxiliary features such as power ridges have been incorporated into aligner designs. Power ridges are small geometric structures integrated into the aligner surface, typically positioned near the gingival third of anterior teeth [16]. These structures aim to increase contact pressure and generate additional moments, thereby improving the transmission of torque to the tooth [17, 18].

More recently, advances in digital orthodontics have enabled the development of directly printed aligners, which represent an alternative to conventional thermoformed appliances. Unlike thermoformed aligners fabricated from thermoplastic sheets, directly printed aligners are manufactured using additive manufacturing technologies such as masked stereolithography apparatus (MSLA) and digital light processing (DLP), depending on the printing system employed [19, 20]. These materials have been designed to exhibit improved mechanical characteristics, including enhanced elasticity, shape memory behaviour, and more consistent thickness distribution [21].

The “program–fix–recover” cycle represents the fundamental thermomechanical mechanism of shape memory polymers (SMPs) [22]. During the programming phase, the material is heated above its transition temperature (Tg) and deformed into a temporary shape. In the fixation phase, cooling below Tg reduces molecular mobility and stabilises the programmed geometry [23, 24]. Upon reheating, the material enters the recovery phase, returning toward its original configuration and generating controlled force delivery [22, 23]. This temperature-responsive behaviour enables sustained and gradual orthodontic force application, making SMP-based materials particularly suitable for directly printed aligner systems [25] .

Despite these technological advancements, limited experimental evidence is available regarding the biomechanical performance of directly printed aligners in delivering torque, particularly when combined with power ridge design features. Therefore, the aim of this in vitro study was to compare the biomechanical efficiency of power ridges in generating buccolingual torque of the maxillary central incisors using thermoformed and directly printed clear aligners in two temperature conditions (25 °C and 37 °C). Torque expression, root and crown displacement, and the associated force moment systems were evaluated using a controlled experimental setup. The null hypothesis was that no significant differences would exist in torque expression, force moment characteristics, or crown and root displacement among the tested aligners in either temperature condition.

## Methodology

### Study design

This investigation was designed as a controlled in vitro experimental study to evaluate and compare the biomechanical efficiency of power ridges in producing buccolingual torque of the maxillary central incisors using different clear aligner fabrication techniques. Two manufacturing approaches were assessed: conventional thermoformed aligners and directly printed aligners. The primary outcome was torque expression, while the secondary outcomes were forces and moments generated during simulated tooth movement, as well as linear root apex and crown displacement.

Four experimental groups were defined based on material type, fabrication technique, and the presence of a power ridge feature. Group ZF-PR consisted of Zendura Flex aligners fabricated via thermoforming with a power ridge. Group ZF-C served as the control, using the same Zendura Flex thermoformed material but without a power ridge. Group TA-28 included directly printed aligners produced from Tera Harz TA-28 with a power ridge. Group DCA comprised directly printed aligners produced from LuxCreo DCA, also incorporating a power ridge (Table 1). All aligners were fabricated from the same STL base models to ensure standardisation of geometry and tooth position.

### Sample size calculation

Sample size calculation was performed using G*Power software (version 3.1; Heinrich Heine University, Düsseldorf, Germany) at a significance level (**α**) of 0.05 and a study power of 80%. Effect size was estimated based on data from a previous biomechanical simulation study [26] evaluating torqueing forces on maxillary central incisors with clear aligners. The minimum required sample size was calculated to be five specimens per group; however, ten samples per group (*n* = 10) were included to enhance statistical robustness and improve estimate precision. The test groups were evaluated under two temperature conditions (25 °C and 37 °C).

### Model preparation

A maxillary virtual dental arch model derived from an anonymised intraoral scan (Primescan, Dentsply Sirona, North Carolina, USA) of a well-aligned dentition was digitally modified using OnyxCeph software (version 3.2.180, Build 492; Image Instruments GmbH, Chemnitz, Germany) to incorporate a 6° lingual root torque adjustment of the right maxillary central incisor (Tooth #11) to simulated a clinical scenario requiring torque correction of the maxillary central incisor. The 6 degrees lingual torque was chosen to represent a clinically relevant magnitude of incisor root correction while producing a measurable biomechanical response within the operating range of the used testing device.

The models were fabricated using a high-resolution 3D printer (P20+; Straumann AG, Basel, Switzerland), from P Pro resin (Straumann AG, Basel, Switzerland). The models were used for the experimental setup and for thermoforming of the ZF-C group. For the ZF-PR group, the power ridge geometry was incorporated into a modified model prior to thermoforming. For the directly printed groups (TA-28 and DCA), the power ridges were incorporated directly into the digital STL file and fabricated as an integral part of the aligner in a single printing step.

**Fig. 1:**
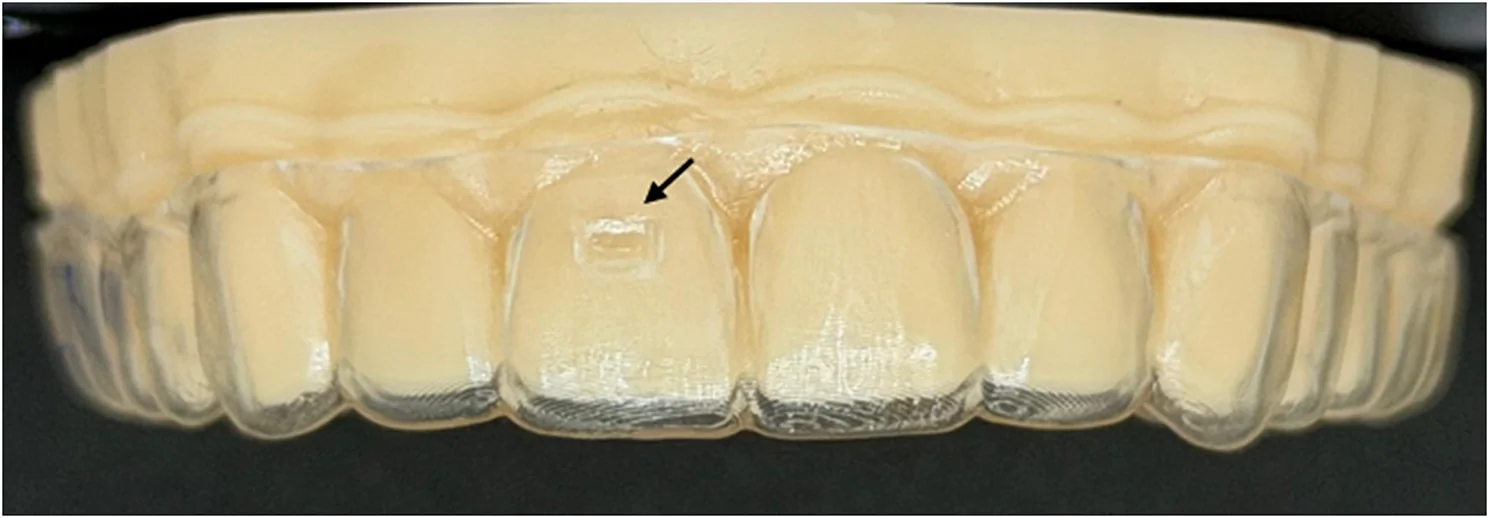
Experimental configuration demonstrating aligner placement on the dental model prior to testing, with buccal power ridge (black arrow) at the maxillary central incisors and a straight trim line 0.5 mm cervical to gingival margin

Power ridges were designed as wedge-shaped indentation with standardised dimensions (1.5 mm height, 3.5 mm width, and 0.8 mm depth) and positioned on the labial surface at the gingival third of tooth #11 following standardised clinical recommendations. The trim line was standardised at 0.5 mm above the gingival margin with a straight cut (Fig. 1).

**Table 1:** Materials and fabrication techniques of thermoformed and directly printed aligner groups

Group	Power ridge	Manufacturer	Fabrication technique	Thickness	Material Type
**ZF-C** (Zendura FLX control group)	**Absent**	**Bay Materials** (Fremont, CA, USA)	Thermoforming was performed using a pressure-forming device (Biostar^®^; Scheu-Dental GmbH, Iserlohn, Germany) at 220 °C for 50 s, followed by pressure application at 5.8 bar and a cooling phase of 60 s.	Original thickness of the sheets 0.75 mm, which decreased to an average of 0.60 mm following the thermoforming process.	Three-layer thermoplastic polyurethane (TPU) sheet consisting of a soft elastomeric middle layer between two hard copolyester outer layers.
**ZF-PR (**Zendura FLX with Power ridge)	**Present**				
**TA-28** **(**Tera Harz TA-28)	**Present**	**Graphy** AG (Seoul, South Korea)	3D printing was performed using masked stereolithography apparatus (MSLA) (Uniz NBEE; Uniz, CA, USA) at a layer thickness of 100 μm. Post-processing comprised centrifugal cleaning (Tera Harz Spinner V2; Graphy Inc., Seoul, South Korea), followed by nitrogen-assisted UV post-curing (Tera Harz Cure; Graphy Inc., Seoul, South Korea) at 405 nm for 25 min, and final immersion in boiling water for 2 min.	0.6 mm	Urethane acrylate oligomer containing acrylic monomers.
**DCA** **(**LuxCreo DCA)	**Present**	**Luxcreo** (Bellevue, WA, USA)	3D printing was performed using the iLux Pro Dental printer (LuxCreo Inc., Bellevue, WA, USA), followed by two-stage isopropyl alcohol cleaning (4 + 4 min; IPA > 95%) using iLuxWash Dental, thermal post-treatment at 130 °C for 10 min using LuxOven Pro, and UV post-curing for 20 min using iLuxCure Pro.	0.6 mm	Photopolymerisable polyurethane-based resin.

### Aligner fabrication

Thermoformed aligners were fabricated using Zendura Flex sheets with a nominal thickness of 0.75 mm. The sheets were pressure formed over the resin dental models using a vacuum/pressure thermoforming unit Ministar S (Scheu-Dental GmbH, Iserlohn, Germany). Following thermoforming, the aligner thickness was reduced approximately from 0.75 mm to around 0.6 mm. Thermoformed aligners were trimmed and finished according to standard laboratory procedures.

### Experimental apparatus

**Fig. 2:**
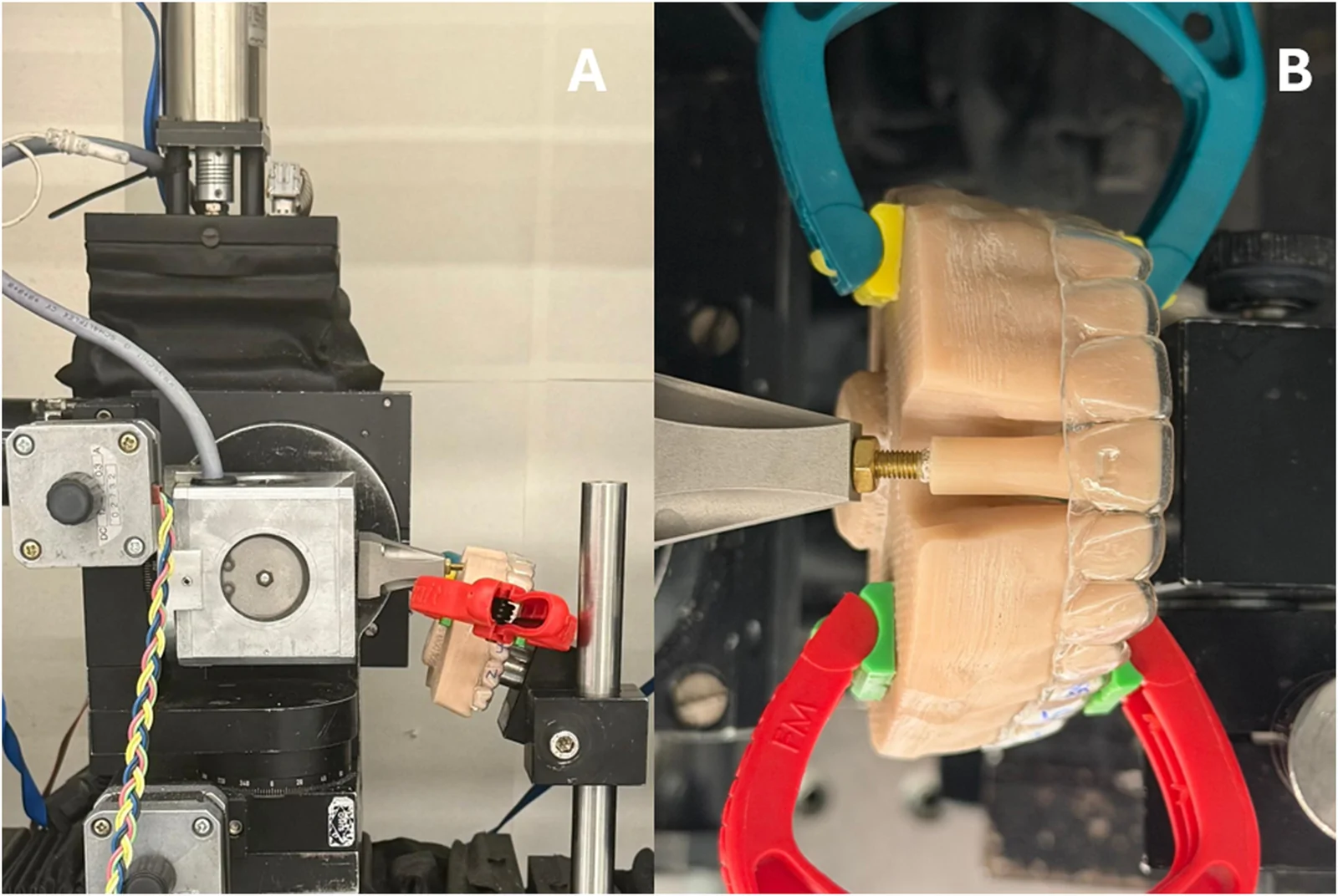
The orthodontic measurement and simulation system (OMSS) (**A**) Lateral view (**B**) Close up view

Three-dimensional force/moment measurements were conducted using the Orthodontic Measurement and Simulation System (OMSS; Oral Technology, University Hospital Bonn, Bonn, Germany) [27]. The OMSS consists of two measurement tables, each comprising a motorised positioning system and a force/torque sensor. The positioning stages are driven by stepping motors, achieving a theoretical positioning resolution of better than 1 μm and 0.001°, respectively. The force/torque sensor is an in-house development with a measurement range of ± 15 N and ± 450 Nmm, and a resolution of 0.02 N for forces and 0.5 Nmm for moments. The accuracy for forces and moments was determined to be 0.02 N and 0.9 Nmm, respectively. The sensor is controlled by custom-developed electronics equipped with a 12-bit analog-to-digital converter (Analog Devices AD574A) featuring a sampling rate of 30 kHz. This enables a complete 6-component force/torque vector—including signal processing and averaging—to be transmitted to the host control computer at a rate of at least 10 Hz [27]. For the measurement task at hand, only one of the two OMSS measurement stages is required to simulate orthodontic tooth displacement under controlled conditions (Fig. 2).

The test model was fabricated in which tooth #11 was digitally subtracted, after which the isolated tooth and the remaining model were printed as separate components. The mesial proximal contact of tooth #12 and the distal proximal contact of tooth #21 were slightly ground to provide adequate space around tooth #11, allowing controlled experimental movement without interference from adjacent teeth while preserving the original arch geometry. A silicon index was fabricated using a non-sectioned model printed from the CAD STL file to record the position of tooth #11 in the dental arch. The tooth #11 was rigidly fixed to the sensor component of the OMSS apparatus, then by the help of the silicon index the dental arch model was fitted and secured with a rigid external fixator so that the tooth #11 was aligned according to the CAD position in the dental arch. After insertion of the first aligner, the forces and moments were checked and the OMSS positioning motor was fine-tuned so that all the recorded forces and moments were nullified; this simultaneously defined the passive-fit starting position for each simulation run. This reference position was saved within the OMSS system, and the tooth was returned to it after each measurement to ensure an accurate and reproducible starting position for all simulations. Additionally, after every aligner insertion and prior to testing, any residual force or moment detected by the sensor was nullified before data recording began. The sensor axis was positioned in a neutral orientation with respect to the X, Y, and Z axes to ensure accurate force registration.

During the stimulation, the tooth was then moved into a malaligned configuration corresponding to a 6° labial root torque discrepancy, representing the inverse of the programmed torque incorporated into the aligner. The aligner, then deformed as a result of the tooth having moved away from its fitted shape, generated a force and moment system directed toward correcting this discrepancy. Following establishment of the malposition, the OMSS initiated simulation of the tooth movement back toward the programmed 6° lingual position, to evaluate the aligner’s ability to express the programmed torque. Force–displacement and moment–rotation relationships were continuously recorded throughout the experimental procedure.

The simulation continued in the form of movement increments of ~ 0.1° and 0.1 mm at the beginning of simulation (gradually decreased to ≤ 0.01° and ≤ 0.01 mm at the end of simulation) until the sensor detects no further force or moment exerted by the aligner on the tooth, indicating that mechanical equilibrium has been reached and the aligner’s capacity to produce further tooth displacement has been exhausted.

Biomechanical measurements were performed at 25 ± 0.5 °C to represent ambient laboratory conditions and at 37 ± 0.5 °C to simulate the physiological intraoral environment, thereby allowing assessment of the temperature-dependent behaviour of the tested aligner materials. The test apparatus was housed within a thermostat-controlled test chamber (Heraeus Vötsch VEM 03/400, Heraeus Instruments GmbH, Düsseldorf, Germany), and temperature was continuously monitored via a digital thermometer to confirm it remained within the target range throughout testing.

### Measured outcomes

The primary outcome was the amount of torque expressed by the aligner. The OMSS was programmed to simulate 6° of labial root torque of the maxillary central incisor, representing the inverse of the torque incorporated into the aligner design.

Expressed torque (Δθ) was calculated as the difference between the prescribed torque and the residual rotation in Y-axis measured at the final simulation step by the OMSS **(R)**:

$$\boldsymbol{\Delta \theta = 6^\circ - R}$$

Secondary outcomes included:

- Force **(F)** and moment **(M)**, which were extracted directly from the OMSS output.
- Root apex and crown displacements along the Y-axis were derived using a geometric lever-arm model based on rotation around the centre of resistance (CRes).

Within the OMSS output, **T** represented translational displacement of the crown reference point along the Y-axis. However, because this value reflects movement of the reference point only, true crown and root apex displacement required incorporation of the rotational component around the CRes.

Root apex displacement **(Apex_Y)** was calculated by accounting for rotational movement using the CRes-to-apex lever arm **(d_apex).** During positive root torque, the root apex moves in the direction opposite to the crown reference point; therefore, the rotational component is subtracted:

$$\boldsymbol{Apex\_Y = T - sin{\text{ }}\left( {\Delta \theta } \right) \times d\_apex}$$

Crown displacement **(Crown_Y)** was calculated by incorporating the rotational component using the crown reference point-to-CRes distance **(d_crown)**. As both translational and rotational components act in the same direction at the crown level during positive root torque, the rotational term is added:

$$\boldsymbol{Crown\_Y = T + sin{\text{ }}\left( {\Delta \theta } \right) \times d\_crown}$$

### Statistical analysis

Five biomechanical variables were extracted from each OMSS simulation output. The primary endpoint was torque expression (°), while secondary outcomes included force (F), moment (M), and linear displacements (Apex Y and Crown Y). Descriptive statistics were calculated for all variables. Data normality was assessed using the Shapiro–Wilk test. Temperature comparisons (25 °C vs. 37 °C) were performed using independent-samples t-tests for normally distributed data or Mann–Whitney U tests for non-normally distributed data, as appropriate. The material effect within each temperature was analyzed using the Kruskal–Wallis test followed by Dunn–Bonferroni post-hoc analysis when indicated. Statistical significance was set at α = 0.05. Analyses were performed using IBM SPSS Statistics for Windows, Version 29.0 (IBM Corp., Armonk, NY, USA).

## Results

### Primary outcome: Torque expression (°)

At 25 °C, DCA demonstrated the highest mean torque expression (3.7 ± 0.8°), followed by ZF-PR (2.5 ± 0.8°), ZF-C (2.3 ± 0.5°), and TA-28 (1.0 ± 0.4°). Torque expression decreased at 37 °C across all materials. However, only DCA exhibited a statistically significant temperature-dependent reduction (independent-samples t-test, *p* = 0.017). No significant temperature-related differences were observed for ZF-PR (*p* = 0.106), ZF-C (*p* = 0.521), or TA-28 (*p* = 0.056), although numerical reductions were observed in all groups (Table 2).

**Fig. 3:**
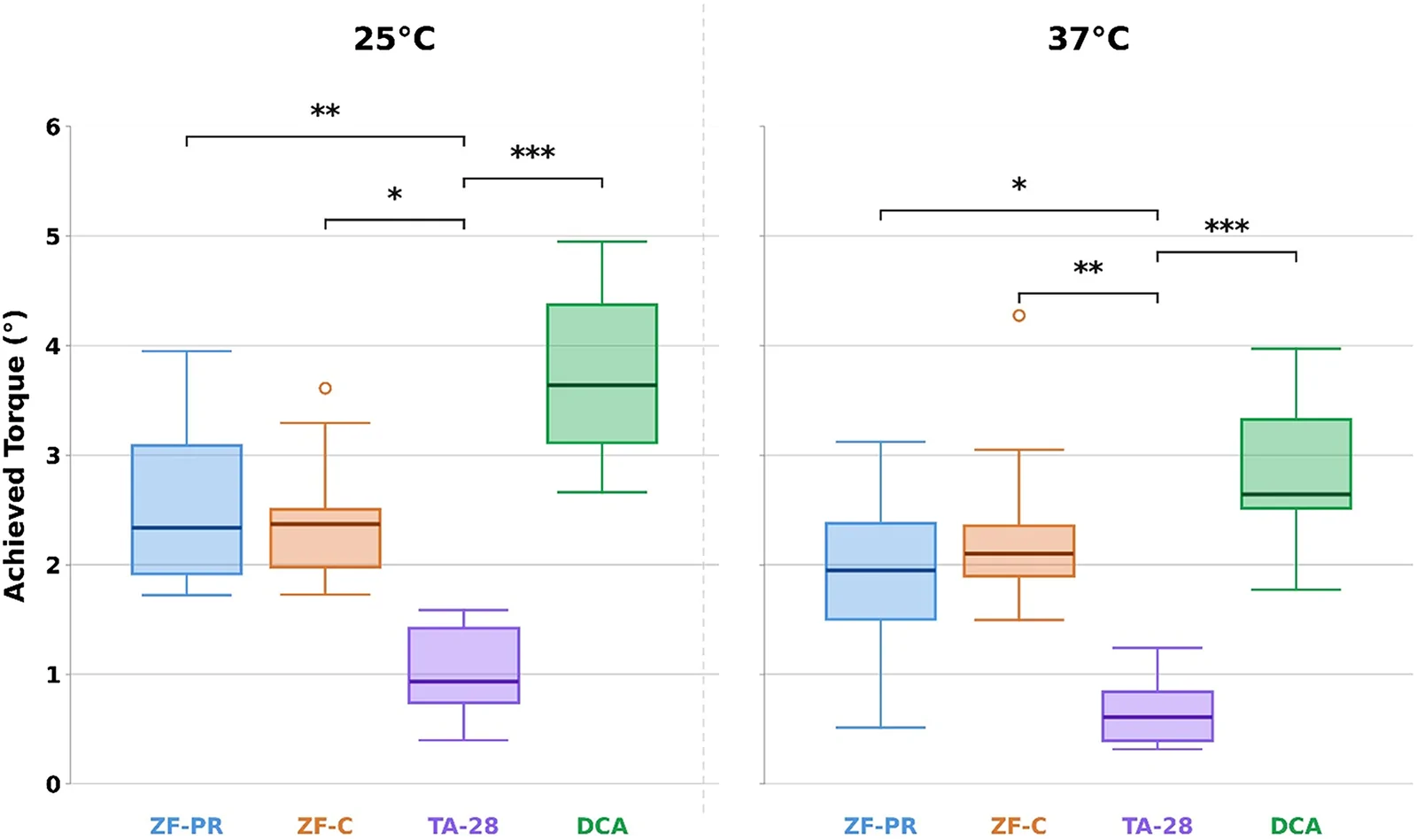
Torque expression (°) at 25 °C (left panel) and 37 °C (right panel) for each aligner material. Significant between-material differences were identified using Dunn–Bonferroni post-hoc analysis following a significant Kruskal–Wallis test. *p < 0.05, **p < 0.01, ***p < 0.001

Significant differences in torque expression were identified among materials at both temperatures (Kruskal–Wallis, *p* < 0.001). At 25 °C, Dunn–Bonferroni post-hoc analysis demonstrated significantly lower torque expression in TA-28 compared with ZF-PR (*p* = 0.007) and ZF-C (*p* = 0.025), while DCA showed significantly greater torque expression than TA-28 (*p* < 0.001). Similar findings were observed at 37 °C, where TA-28 remained significantly lower than ZF-PR (*p* = 0.042) and ZF-C (*p* = 0.004), and DCA continued to demonstrate significantly greater torque expression than TA-28 (*p* < 0.001). No significant differences were detected between DCA and ZF-PR at either temperature. The between-material comparisons are illustrated in Fig. 3.

### Secondary outcomes

#### Force (N)

At 25 °C, mean force values ranged from − 0.12 ± 0.14 N (TA-28) to − 0.25 ± 0.17 N (DCA), with all materials exhibiting lingually directed forces. No significant temperature-related differences were observed for ZF-PR (*p* = 0.266) or ZF-C (*p* = 0.745). In contrast, TA-28 demonstrated a significant temperature-dependent change in force direction, shifting from − 0.12 ± 0.14 N at 25 °C to + 0.02 ± 0.05 N at 37 °C (Mann–Whitney U, *p* = 0.001). DCA also showed a significant reduction in lingual force magnitude at 37 °C (*p* < 0.001) **(**Table 2**).**

**Fig. 4:**
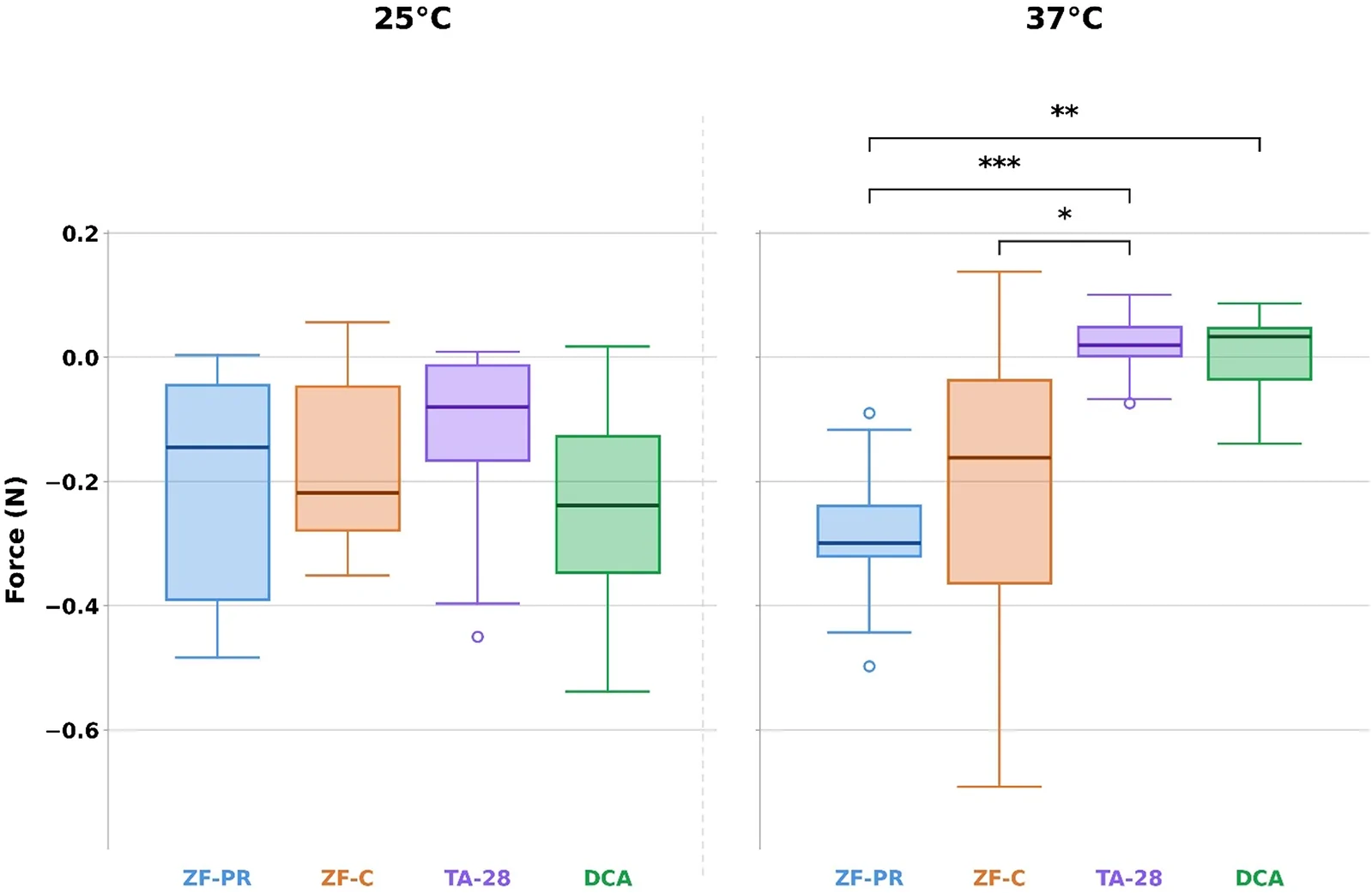
Force (N) at 25 °C (left panel) and 37 °C (right panel) for each aligner material (*n* = 10 per group). Negative values indicate a lingual force direction while positive values indicate a labial force direction. Significant pairwise post-hoc differences were identified using Dunn–Bonferroni analysis following a significant Kruskal–Wallis test. * *p* < 0.05; ** *p* < 0.01; *** *p* < 0.001

No significant between-material differences in force were identified at 25 °C (Kruskal–Wallis, *p* = 0.395). However, significant differences emerged at 37 °C (Kruskal–Wallis, *p* < 0.001). Dunn–Bonferroni post-hoc analysis demonstrated significant differences between ZF-PR and TA-28 (*p* < 0.001), ZF-PR and DCA (*p* = 0.003), and ZF-C and TA-28 (*p* = 0.046). Between-material comparisons are illustrated in Fig. 4.

#### Moment

Moment values were negative across all groups at both temperatures, indicating a consistent lingual bending moment. At 25 °C, DCA generated the greatest moment magnitude (− 23.3 ± 3.5 N·mm), followed by ZF-PR (− 17.5 ± 3.9 N·mm), ZF-C (− 13.5 ± 4.1 N·mm), and TA-28 (− 3.9 ± 1.1 N·mm). A significant reduction in moment magnitude at 37 °C was observed for ZF-PR (*p* = 0.003) and DCA (*p* = 0.030), whereas ZF-C (*p* = 0.645) and TA-28 (*p* = 0.060) showed no significant temperature-related change (Table 2).

**Fig. 5:**
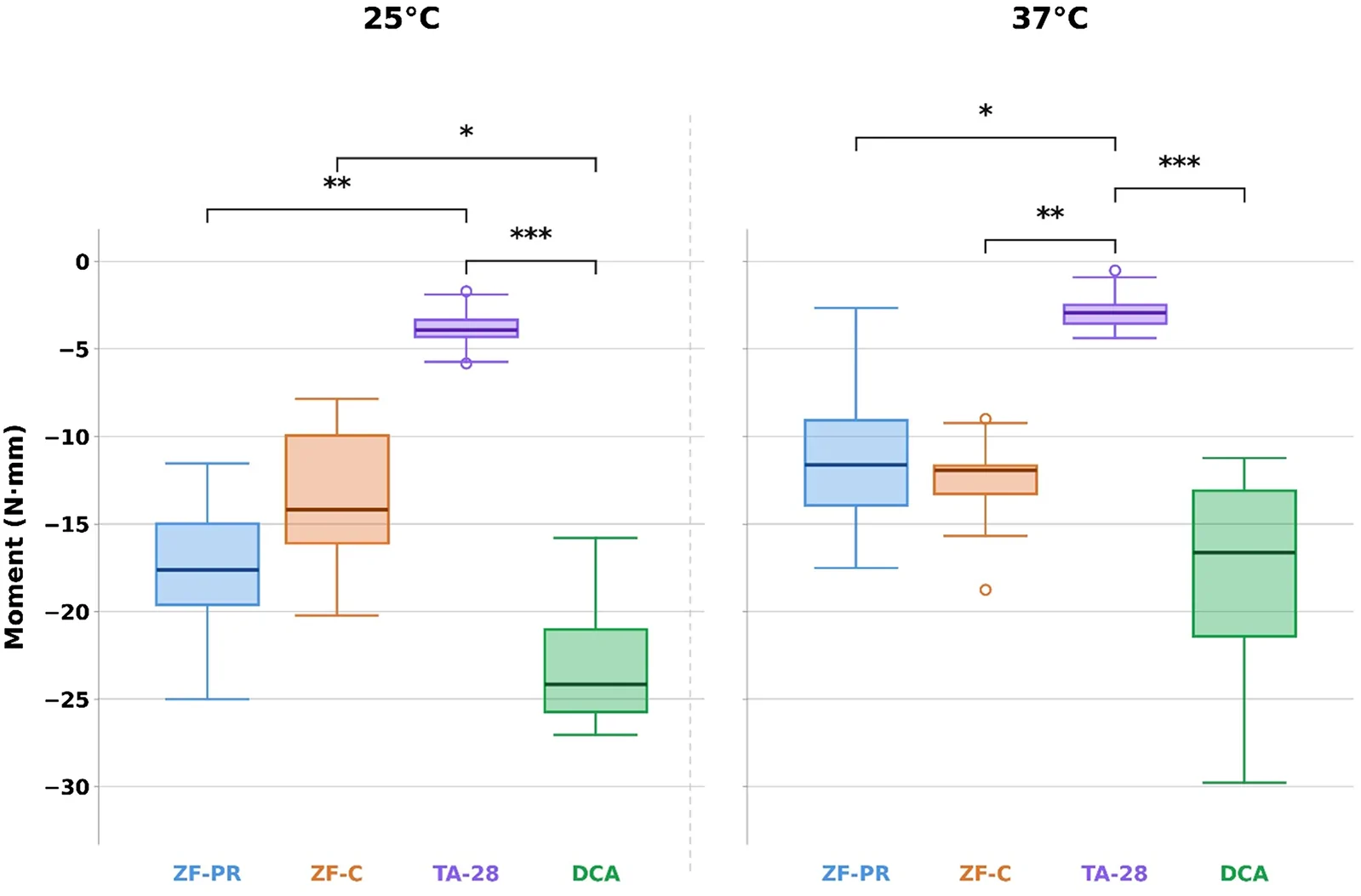
Moment (N·mm) at 25 °C (left panel) and 37 °C (right panel) for each aligner material. Negative values indicate clockwise moment while positive values indicate counterclockwise moment. Significant pairwise post-hoc differences were identified using Dunn–Bonferroni analysis following a significant Kruskal–Wallis test. * *p* < 0.05; ** *p* < 0.01; *** *p* < 0.001

Significant between-material differences in moment were detected at both temperatures (Kruskal-Wallis *p* < 0.001). At 25 °C, TA-28 generated significantly lower moments than ZF-PR (*p* = 0.002) and DCA (*p* < 0.001), while ZF-C also produced lower moments than DCA (*p* = 0.019). At 37 °C, TA-28 remained significantly lower than ZF-PR (*p* = 0.035) and ZF-C (*p* = 0.004), whereas DCA continued to demonstrate significantly greater moment generation than TA-28 (*p* < 0.001). No significant difference was identified between ZF-PR and DCA at either temperature. Moment comparisons are presented in Fig. 5.

#### Root apex displacement

Root apex displacement values are presented as absolute magnitudes, with negative values in the raw OMSS output corresponding to lingual root movement. At 25 °C, DCA demonstrated the greatest apex displacement (1.0 ± 0.2 mm), followed by ZF-PR (0.5 ± 0.2 mm), ZF-C (0.5 ± 0.2 mm), and TA-28 (0.3 ± 0.1 mm). Among the tested materials, only DCA showed a significant reduction in apex displacement at 37 °C (*p* = 0.026), decreasing to 0.7 ± 0.3 mm. No significant temperature-related differences were observed for ZF-PR (*p* = 0.648), ZF-C (*p* = 0.835), or TA-28 (*p* = 0.226) **(**Table 2**).**

**Fig. 6:**
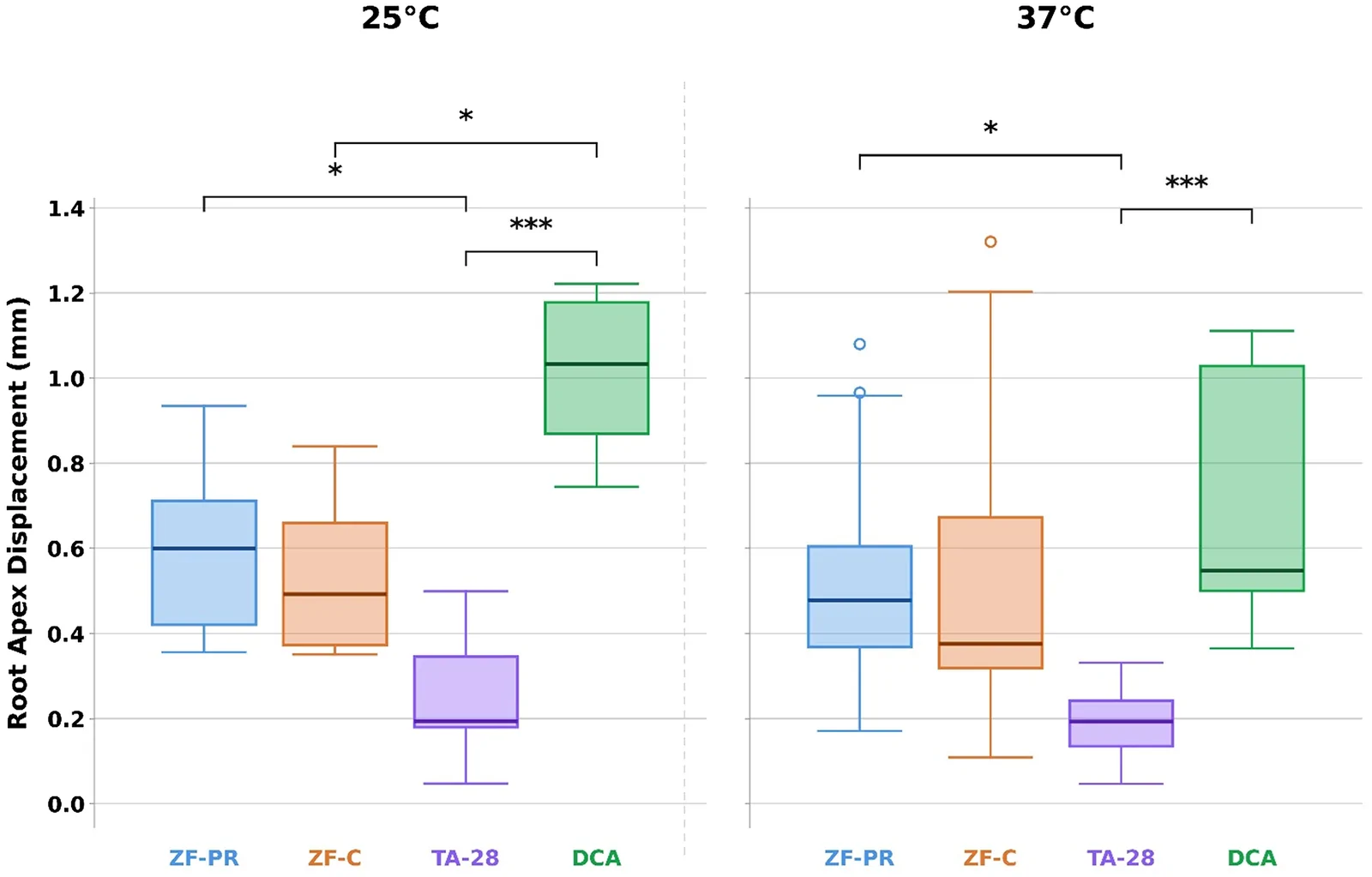
Root apex displacement (mm) at 25 °C (left panel) and 37 °C (right panel) for each aligner material. Significant pairwise post-hoc differences were identified using Dunn–Bonferroni analysis following a significant Kruskal–Wallis test. *p < 0.05; **p < 0.01; **p < 0.001

Significant between-material differences in root apex displacement were identified at both temperatures (Kruskal–Wallis, *p* < 0.001). At 25 °C, DCA demonstrated significantly greater apex displacement than ZF-C (*p* = 0.011) and TA-28 (*p* < 0.001), while ZF-PR also exceeded TA-28 (*p* = 0.047). At 37 °C, TA-28 remained significantly lower than both ZF-PR (*p* = 0.010) and DCA (*p* < 0.001). No significant differences were detected among ZF-PR, ZF-C, and DCA at 37 °C. Root apex displacement comparisons are presented in Fig. 6.

#### Crown displacement

At 25 °C, crown displacement ranged from 0.2 ± 0.1 mm (TA-28) to 0.7 ± 0.2 mm (DCA). Significant reductions in crown displacement at 37 °C were observed for ZF-PR (*p* = 0.012) and TA-28 (*p* = 0.021). In ZF-PR, crown displacement decreased from 0.6 ± 0.2 mm to 0.4 ± 0.2 mm, whereas TA-28 showed a marked reduction from 0.2 ± 0.1 mm to 0.1 ± 0.1 mm. No significant temperature-related differences were detected for ZF-C (*p* = 0.910) or DCA (*p* = 0.146), although numerical reductions were observed in both groups (Table 2).

**Fig. 7:**
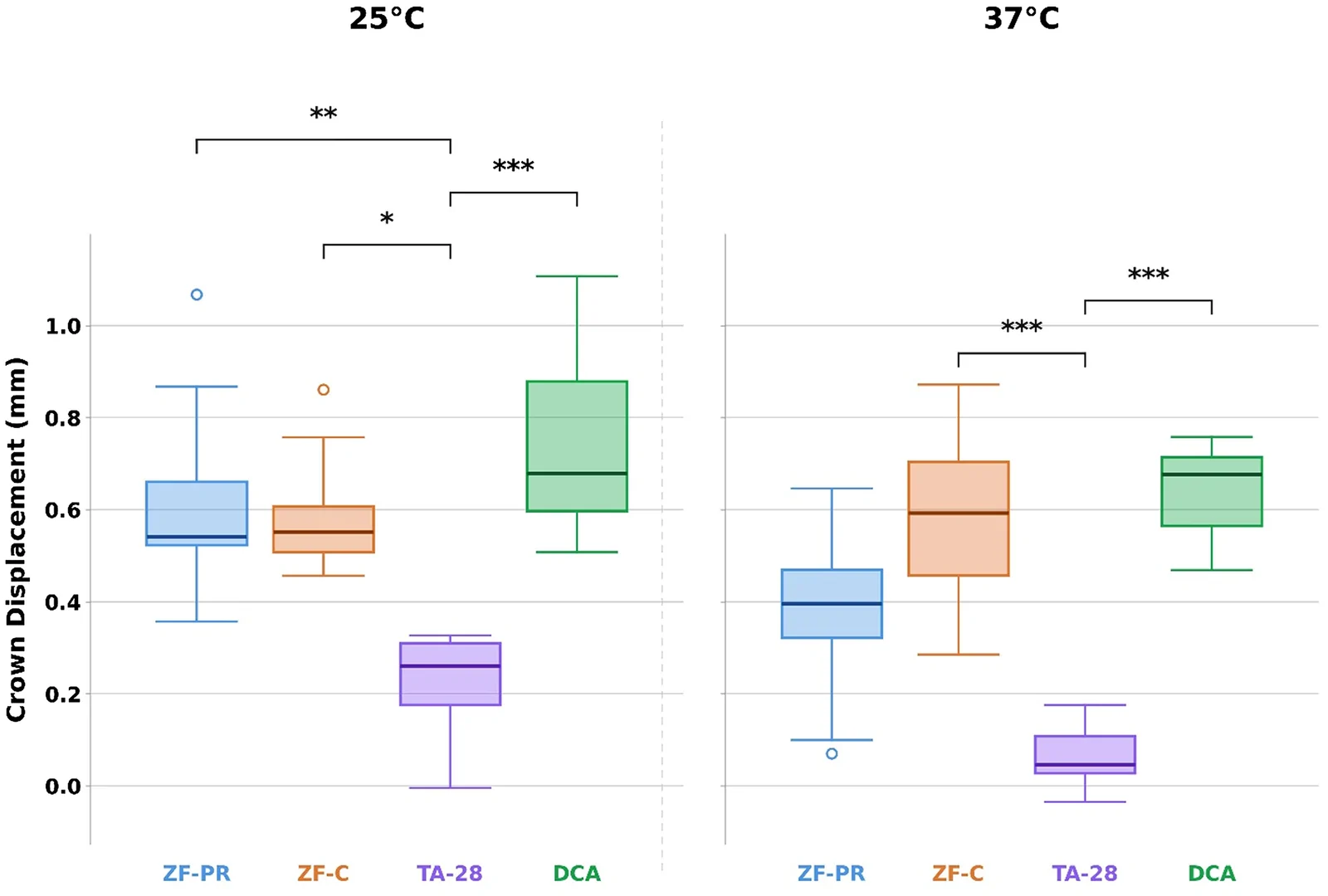
Crown Displacement (mm) at 25 °C (left panel) and 37 °C (right panel) for each aligner material. Significant pairwise post-hoc differences were identified using Dunn–Bonferroni analysis following a significant Kruskal–Wallis test. *p < 0.05; **p < 0.01; ***p < 0.001

Significant between-material differences in crown displacement were identified at both 25 °C and 37 °C (Kruskal–Wallis, *p* < 0.001). At 25 °C, TA-28 demonstrated significantly lower crown displacement than ZF-PR (*p* = 0.003), ZF-C (*p* = 0.010), and DCA (*p* < 0.001). No significant differences were observed among ZF-PR, ZF-C, and DCA at 25 °C. At 37 °C, TA-28 remained significantly lower than both ZF-C (*p* < 0.001) and DCA (*p* < 0.001), whereas no significant differences were identified among ZF-PR, ZF-C, and DCA. Crown displacement comparisons are presented in Fig. 7. Each material was tested at 25 °C and 37 °C with *n* = 10 simulations per subgroup. An overview of between-temperature comparisons across all variables and materials is presented in Table 2.

**Table 2:** Pairwise comparisons of biomechanical outcomes between 25 °C and 37 °C for each aligner material (ZF-PR, ZF-C, TA-28, and DCA)

Outcome	ZF-PR		ZF-C		TA-28		DCA	
	Statistical Test	*p*-value	Statistical Test	*p*-value	Statistical Test	*p*-value	Statistical Test	*p*-value
**Achieved Torque (°)**	t-test	0.1056 ns	Mann-Whitney U	0.5205 ns	t-test	0.0555 ns	t-test	0.0167 *
**Force (N)**	t-test	0.2655 ns	t-test	0.7446 ns	Mann-Whitney U	0.0013 **	t-test	0.0005 ***
**Moment (N·mm)**	t-test	0.0030 **	t-test	0.6446 ns	t-test	0.0596 ns	t-test	0.0295 *
**Root Apex Displacement (mm)**	t-test	0.6477 ns	t-test	0.8346 ns	t-test	0.2262 ns	Mann-Whitney U	0.0257 *
**Crown Displacement (mm)**	t-test	0.0118 *	Mann-Whitney U	0.9097 ns	Mann-Whitney U	0.0211 *	t-test	0.1462 ns

*p < 0.05, **p < 0.01, ***p < 0.001

## Discussion

In the present study, the torque expression, force, moment, and crown/root displacement varied significantly among the tested groups in both temperature conditions, therefore the null hypothesis was rejected. The torque expression was identified in the current study as the principal differentiating outcome among the tested aligner systems. Using ZF-C as the baseline reference, the results showed a relatively consistent but only moderate level of expressed torque across temperature conditions, whereas DCA demonstrated the highest torque expression and the clearest temperature responsiveness. In contrast, TA-28 consistently exhibited the lowest torque expression and the weakest thermal response. This overall pattern is clinically relevant because torque has repeatedly been identified as one of the least predictable tooth movements during clear aligner therapy, particularly when root control is required [14]. Clinical CBCT-based studies have reported that incisor torque is under expressed relative to the programmed movement, with one study reporting mean torque efficacy of 35.21%, while another found that clear aligners tend to lose upper incisor torque and may require overcorrection to compensate for reduced root movement [9].

Against this background, the present findings suggest that ZF-C behaves as a useful baseline material because it provided comparatively consistent torque expression across 25 °C and 37 °C, despite its moderate overall performance. Clinically, this behaviour may be interpreted as predictable but limited torque efficiency, indicating that conventional thermoformed aligners may still require programmed overcorrection or auxiliary features when substantial root movement is needed [28]. This interpretation is consistent with the broader literature showing that conventional clear aligner biomechanics often produce less root movement than planned, even when crown movement appears acceptable [18].

DCA demonstrated the highest torque expression under both temperature conditions. From a biomechanical perspective, torque is governed by the generation and maintenance of an appropriate force moment system rather than force magnitude alone [14]. The superior performance of DCA therefore suggests more efficient generation and transmission of the force–moment system required to produce controlled root movement. These characteristics support more efficient generation and transmission of the force–moment system under intraoral conditions, thereby enhancing torque expression. In contrast, the limited torque performance observed in TA-28, despite measurable force generation, suggests that **material-specific** limitations in stiffness recovery or stress transmission may impair **the efficient generation and transmission of the force moment system** [29].

Within this framework, the enhanced performance of DCA may be attributed to its temperature responsive viscoelastic properties [30]. Previous studies have shown that TC-85 (Graphy Inc., Seoul, South Korea) exhibits sustained residual force levels, improved strain recovery, and reduced storage modulus at elevated temperatures while maintaining dimensional stability [25]. These characteristics support more efficient generation and transmission of the force moment system under intraoral conditions, thereby enhancing torque expression. In contrast, the limited torque performance observed in TA-28, despite measurable force generation, suggests that material specific limitations in stiffness recovery or stress transmission may the efficient generation and transmission of the force moment system.

The ZF-PR group occupied an intermediate position in this framework. Compared with baseline ZF-C, the addition of power ridges appeared to increase temperature responsiveness. This suggests that design modifications can partially enhance torque delivery even when the base material remains the same. The direction of this finding is in line with prior biomechanical literature showing that auxiliaries such as power ridges and horizontal ellipsoid attachments can improve torque control by modifying how forces and moments are delivered to anterior teeth [7]. However, the inability of ZF-PR to outperform DCA indicates that design modifications alone cannot fully compensate for material dependent limitations in stiffness recovery, force decay, and the efficient generation and transmission of the force moment system.

The present findings regarding TA-28 are consistent with the recent in vitro investigation by Cho et al., [31] who reported that Graphy TA-28 generated lower force and moment magnitudes compared with thermoformed aligners and demonstrated unexpected force directions during labial tipping mechanics. Specifically, the authors observed that TA-28 produced lingual forces despite programming labial tipping movement and consistently generated the lowest absolute moment values among the tested materials. Furthermore, at greater activation levels, TA-28 exhibited significantly weaker labial inclination moments than thermoformed materials, suggesting reduced biomechanical efficiency in transmitting rotational forces. These observations closely parallel the present results, in which TA-28 generated measurable force but limited torque expression, reduced moment generation, and minimal apical displacement. The low biomechanical output of TA-28 especially at 37 °C is attributed to its shape memory properties, which manifest as thermal softening beyond the working range and viscoelastic springback during insertion of aligner on the model within the relatively short duration of the OMSS simulation. DCA does not exhibit the same behaviour despite also being a shape memory material, as its glass transition temperature is substantially higher than that of TA-28 [30].

The accompanying moment, apex displacement, and crown displacement findings support this interpretation. Compared with DCA, TA-28 demonstrated weaker displacement patterns and reduced evidence of effective root movement. Clinically, this would be expected to translate into reduced torque efficiency and an increased tendency toward uncontrolled tipping, consistent with previous reports identifying root torque as one of the most difficult movements to achieve predictably with aligners [25].

The secondary biomechanical outcomes in the present study were essential for interpreting the torque findings. Among these variables, moment generation was the most directly associated with torque expression because it represents the rotational component required for root movement. Apex and crown displacement patterns further clarified whether the observed response reflected true torque or simple crown tipping. In this regard, the superior performance of DCA appeared biomechanically coherent, with greater torque expression accompanied by displacement patterns consistent with controlled root movement. Conversely, the weaker secondary outcome profile of TA-28 reinforced the conclusion that its limited torque expression reflected biomechanical inefficiency rather than insufficient force magnitude alone [14].

From a clinical perspective, these findings suggest a practical hierarchy among the tested systems. ZF-C may be considered a consistent baseline material with moderate torque capability, DCA demonstrated the most favourable biomechanical profile for clinically demanding torque movements, and TA-28 showed limited effectiveness despite measurable force generation. More broadly, the present findings reinforce a key principle of aligner biomechanics: successful torque control depends less on nominal programmed displacement and more on the aligner system’s ability to generate and sustain an appropriate force moment system [25].

Taken together, the present results suggest that differences in torque expression among aligner systems are biomechanically meaningful rather than merely numerical, and that secondary measures such as moment generation and apical displacement are essential for explaining why some materials achieve clinically useful torque while others do not.

As an in vitro investigation, this study has some limitations such as the inability of the experimental setup to fully replicate the biological and biomechanical complexities of the clinical environment, including periodontal ligament behavior, occlusal interactions, and other intraoral conditions such as saliva, humidity, and continuous thermal fluctuations. In addition, only a single programmed torque movement (6° lingual root torque) of one maxillary central incisor was evaluated, limiting the generalisability of the findings to other tooth movements and clinical scenarios. Therefore, further in vivo and long-term investigations are warranted to validate the present findings before clinical extrapolation.

## Conclusion

Within the limitations of this in vitro study, torque expression was found to be strongly influenced by aligner material properties and thermomechanical behavior. DCA demonstrated the highest torque expression, characterised by greater moment generation and increased apical displacement, indicative of effective root movement and true torque expression. In contrast, TA-28 exhibited limited torque expression despite measurable force generation, accompanied by reduced moment and minimal apical displacement, suggesting inefficient generation and transmission of the force–moment system required for effective root movement. ZF-C demonstrated consistent but moderate torque performance, whereas the incorporation of power ridges in ZF-PR provided only modest enhancement in torque delivery. Collectively, these findings indicate that effective torque control in clear aligner therapy depends not on force magnitude alone, but on the material’s ability to efficiently generate and sustain an appropriate force–moment system under clinically relevant conditions.

## Clinical interpretation

Clinically, torque expression varied significantly among materials, directly affecting the predictability of anterior tooth movement. ZF-C demonstrated consistent but moderate performance, suggesting that auxiliary features or overcorrection may still be required for effective root control. In contrast, DCA showed superior torque expression and greater apical displacement, indicating improved root movement efficiency. TA-28 exhibited limited torque expression despite measurable force generation, suggesting inefficient generation and transmission of the force–moment system and a greater tendency toward uncontrolled tipping. These findings highlight that effective torque control depends primarily on the material’s ability to generate and sustain an appropriate force–moment system rather than on force magnitude alone.

## Data Availability

The datasets generated and analysed during the current study are available from the corresponding author upon reasonable request.

## Abbreviations

CAT: Clear Aligner Therapy
CAD: Computer Aided Design
OMSS: Orthodontic Measurement and Simulation System
DLP: Digital Light Processing
STL: Standard Tessellation Language
TPU: Thermoplastic Polyurethane
SMP: Shape Memory Polymer
Tg: Glass Transition Temperature
F: Force in the y-axis
M: Moment in the y-axis
T: Translation in the y-axis
R: Rotation around the y-axis
Apex Y: Apical displacement along the y-axis
Crown Y: Crown displacement along the y-axis
ZF-C: Zendura Flex control (thermoformed, no power ridge)
ZF-PR: Zendura Flex with power ridge
TA-28: Tera Harz TA-28 (Graphy)
DCA: LuxCreo DCA (LuxCreo)
